# *In Vivo* Imaging of Transplanted Pancreatic Islets

**DOI:** 10.3389/fendo.2017.00382

**Published:** 2018-01-22

**Authors:** Donghee Kim, Hee-Sook Jun

**Affiliations:** ^1^Lee Gil Ya Cancer and Diabetes Institute, Gachon University, Incheon, South Korea; ^2^College of Pharmacy, Gachon Institute of Pharmaceutical Science, Gachon University, Incheon, South Korea; ^3^Gachon Medical Research Institute, Gil Hospital, Incheon, South Korea

**Keywords:** pancreatic islet, non-invasive imaging, islet mass, magnetic resonance imaging, positron emission tomography, optical imaging

## Abstract

The beta-cells in the islets of Langerhans in the pancreas secrete insulin and play an important role in glucose homeostasis. Diabetes, characterized by hyperglycemia, results from an absolute or a relative deficiency of the pancreatic beta-cell mass. Islet transplantation has been considered to be a useful therapeutic approach, but it is largely unsuccessful because most of the transplanted islets are lost in the early stage of transplantation. To evaluate the efficacy of intervention methods for the improvement of islet survival, monitoring of the functional islet mass is needed. Various techniques to image and track transplanted islets have been investigated to assess islets after transplantation. In this review, recent progresses in imaging methods to visualize islets are discussed.

## Introduction

The pancreas is composed of endocrine and exocrine tissues. The endocrine pancreas constitutes only 1–2% of the total pancreatic mass and is composed of cell clusters called the islets of Langerhans. The islets of Langerhans contain insulin-producing beta-cells (about 80% of the islet cells), glucagon-producing alpha-cells, somatostatin-producing delta-cells, pancreatic peptide-producing PP-cells, and ghrelin-producing epsilon-cells. The exocrine pancreas, which occupies most of the pancreatic tissue and produces digestive enzymes, is composed of acinar cells and ductal cells. As the islets of Langerhans are scattered sparsely throughout the pancreas, it is difficult to access them for *in vivo* imaging.

Diabetes is characterized by defective control of blood glucose resulting from an absolute or relative deficiency of insulin, the hormone released from pancreatic beta-cells. Type 1 diabetes results from the insufficient production of insulin due to the autoimmune-mediated destruction of pancreatic beta-cells (1, 2). Type 2 diabetes results from insufficient insulin to compensate for insulin resistance (3). Insulin therapy has been clinically used for the treatment of diabetes; however, insulin injections do not restore tight glycemic control or prevent diabetes-associated complications. An alternative and safe method for the treatment of diabetes is islet transplantation, which can restore insulin production by implanting functioning pancreatic islets into the liver of diabetic patients (4). Since the first attempt at clinical islet transplantation in 1974 (5), numerous trials have been performed. However, alloislet transplantation still has obstacles for treatment of type 1 diabetes because of the lack of pancreatic islet donors. Therefore, porcine islets (6) or beta-cells differentiated from stem cells (7, 8) have been investigated as sources of islets for transplantation, but are far from clinical application. In addition, islet rejection due to an inflammatory response called instant blood-mediated inflammatory reaction, hypoxia, nutrient deprivation, and islet toxicity caused by immunosuppressants result in significant islet loss in the early transplantation stage (9, 10).

Monitoring the condition of pancreatic islets would be important to assess the outcome of islet transplantation. The ability to image and track islets and to quantify viable islets after transplantation and correlate this with their function (functional beta-cell mass) before blood glucose changes occur, which is a late marker for islet dysfunction, could guide an appropriate treatment for prevention of islet loss. Endogenous islet imaging is particularly challenging, due to the deep abdominal location, size (50–600 µm in diameter), and density (about 1%) in the pancreas. In addition, a specific probe for beta-cells, but not for other cells such as alpha-, delta-, or exocrine cells, is needed to accurately measure the beta-cells.

Non-invasive monitoring of transplanted islets have been investigated using different imaging modalities such as positron emission tomography (PET), single-photon emission computed tomography (SPECT), magnetic resonance imaging (MRI), ultrasonography (US), bioluminescence imaging (BLI), and fluorescence imaging. In this review, we briefly summarize the recent progress in non-invasive islet imaging of transplanted pancreatic islets.

## Positron Emission Tomography

Positron emission tomography is a non-invasive nuclear medical imaging modality routinely used in the hospital for diagnosis of cancers. PET is much more sensitive than MRI and is able to detect picomoles of positron-emitting tracer (11). Imaging is obtained by the administration of molecules labeled with positron-emitting radioisotopes such as 2-[^18^F] fluoro-2-deoxy-d-glucose (FDG). When islets labeled with FDG were transplanted into the liver of syngeneic rats, PET imaging detected islets up to 6 h after transplantation (12). In addition, preclinical trials in pigs using FDG-labeled porcine islets revealed that about 50% of the infused radioactivity was detected in the liver after intraportal transplantation with no accumulation in the lungs or brain (13), suggesting a potential for clinical application. In 2009, clinical trials were carried out in six patients in which FDG-labeled islets were detected in the liver of patients without adverse effects (14). This was the first report showing the clinical feasibility of FDG/PET imaging for real-time quantitative and qualitative evaluation of pancreatic islets. However, one drawback to FDG is its short half-life, making it difficult to monitor islets for a long time. Several other probes specific to beta-cells have been developed such as [^18^F] dithizon targeting Zn ions in secretory granule of pancreatic islets, [^11^C] dihydrotetrabenazine (DTBZ) targeting vesicular monoamine transporter 2 (VMAT2), [^11^C] and [^18^F] l-3,4-dihydroxyphenylalanine ([^18^F]DOPA) (a catecholamine precursor). Injection of [^11^C] DTBZ visualized islets in normal rats, but not in diabetic rats (15), suggesting that VMAT2 in beta-cells was successfully targeted by this method. Also it was reported that rat islets transplanted into muscle were visualized by DTBZ and PET (16).

The glucagon-like peptide (GLP)-1 receptor agonist, exendin-4, also has been investigated as an effective probe for PET imaging of islets, as the GLP-1 receptor is highly expressed in beta-cells (17, 18). In addition, a study on GLP-1 receptor-based imaging of human beta-cells transplanted into the muscle of patients suggests the feasibility of this method of islet mass measurement in clinical transplantation (19). A recent report also revealed that an imaging marker for islet mass, the serotonin precursor [^11^C]5-hydroxytryptophan, which is clinically used for localization of neuroendocrine metastasis in the liver, showed a positive correlation between the hepatic uptake and function of intraportal transplanted islets (20), suggesting a promising tool for monitoring viable pancreatic islets. Beta-cell-specific peptides and antibodies that can be tagged with PET reporters have been also investigated for PET imaging (21, 22). More recently, *in vivo* PET imaging of viable subcutaneous human islets was conducted using [^18^F]DOPA as a biomarker for the transplanted islet mass (23). However, all of these PET tracers for visualizing transplanted islets use ionizing radiation, so developing PET tracers with non-toxic, high specific binding to beta-cells in the pancreatic islets is an important objective for future studies.

## Single-Photon Emission Computed Tomography

Single-photon emission computed tomography is a nuclear medical imaging modality using gamma rays. SPECT can evaluate islet function based on the tracer enhancement as a marker of radioactivity, but the spatial resolution is not good. Transplanted beta-cells (INS-1 832/13) stably transfected with a herpes simplex virus type 1–thymidine kinase-green fluorescent protein fusion construct could be visualized with 5-^131^I-iodo-1-(2-deoxy-2-fluoro-b-d-arabinofuranosyl) uracil by SPECT imaging in an animal model (24). It was recently reported that SPECT quantification of ^111^In-exendin-3 uptake was positively correlated with the insulin-positive area of islet transplants in the muscle of mice, suggesting potential for *in vivo* monitoring of beta-cell mass in islet grafts (25). Like PET, SPECT tracers are also based on radioactivity, so it is necessary to develop a non-toxic tracer.

## Magnetic Resonance Imaging

Magnetic resonance imaging is an attractive islet imaging modality due to a high spatial resolution, good penetration, longer imaging time than other methods, no ionizing radiation, and repeatable clinical measurements. An MRI requires exogenous contrast agents to enhance visualization of pancreatic islets. Superparamagnetic iron oxide (SPIO) particles have long been used as an MRI contrast agent for imaging, and the MR signal by iron oxide labeling is able to image a single cell. Rat pancreatic islets labeled with SPIO particles were visualized by MRI *in vitro* and also *in vivo* for 22 weeks post-transplantation in an animal model (26). The SPIO labeling of islets did not affect islet cell viability or beta-cell function (27). Transplanted SPIO-labeled islets in the liver were imaged as hypointense spots, which may represent either a single labeled islet or a cluster of many islets (27). These hypointense spots disappeared when the transplanted allogeneic or xenogeneic islets were rejected (28, 29), indicating that SPIO-labeled islets could be visualized, although this method could not monitor islet viability. The SPIO particles modified with the near-infrared fluorescent Cy5.5 dye were used to label human islets and image them after transplantation to the kidney capsule and liver of a mouse (30). The first clinical trial of MRI of SPIO-labeled islets transplanted into the liver has been carried out, but no correlation was observed between number of transplanted islets and the number of spots within the liver (31). Recently, clinical grade iron nanoparticles, ferucarbotran (Resovist^®^), have been tried for labeling islets, and low toxicity and signal stability of this contrast agent were shown (32, 33). Transplanted ferucarbotran-labeled islets in C-peptide-negative patients were visualized up to 24 weeks by MRI, and MR detection was correlated with C-peptide production (34).

To improve contrast enhancement, an MRI contrast agent that uses paramagnetic ions [gadolinium (Gd)] was used to label human islets, and the islets could be visualized when transplanted in immune-deficient mice. The resulting hyperintense spots were easier to identify and quantify than SPIO-labeled cells, as the volume of enhancement was equal to the cell size (35). However, Gd-based agents showed adverse effects in patients with nephrogenic systemic fibrosis. Imaging of perfluorocarbon-labeled human islets using fluorine-19 (^19^F) MRI was reported (36), and this agent has no background ^19^F signal in tissues (37). Most recently, labeling of human islets with multiwalled carbon nanotubes has been investigated for imaging transplanted islets, suggesting that these nanotubes can be an alternative labeling compound to be used with human islets for experimental and transplantation studies (38).

Although their application to transplanted islets have not been reported, Zn- and Mn-targeted MRI techniques might be used for visualizing transplanted islets because these ions reflect insulin production and therefore the mass of beta-cells. Since Mn^2+^ ions enter beta-cells through voltage-gated Ca^2 +^ channels, Mn^2+^ ion-labeled beta-cells can be measured by hyperintensity on MR images (39). The Zn^2+^ ion is secreted together with insulin in response to glucose, therefore MRI detection of Zn^2+^ released from beta-cells is only observed during glucose-stimulated insulin secretion (40).

An MRI is considered as an ideal non-invasive method for transplanted islet cells; however, quantification of the islet mass by contrast agents remains challenging. 3D radial ultrashort echo time (UTE) imaging is proposed as a technique for quantifying the transplanted islet mass (41). Crowe et al. used Resovist (carboxydextran-coated SPIO particles) as a contrast agent, and they found that the images showed quantifiable positive contrast from the labeled cells. The 3D radial UTE method has shown the ability to detect spots clearly and distinctly, as well as assessing and quantifying changes with number of cells and progression over time (42). Further studies are required for clinical application.

## Ultrasonography

Ultrasonography imaging is one of the most used methods for the clinical and diagnostic evaluation of the pancreas due to its safety (no ionizing radiation exposure for the patient), high availability in clinical settings, and relative ease of use. High-frequency US was used to image islets transplanted into subrenal capsule of diabetic mice, and the calculated islet volume was positively correlated with the number of transplanted islets and serum insulin levels (43). US imaging has been tried in the clinical setting and could detect the aggregate of islets, but not individual transplanted islets, in the portal vein during transplantation (44), indicating the possible use of US for imaging transplanted islets. Further studies are required for the clinical application.

## Bioluminescence and Fluorescence Imaging

Bioluminescence imaging is an optical technique for imaging islets using light-generating enzymes (e.g., luciferase reporter gene). The transfected reporter gene in cells or the transgenic expression of the reporter gene in an animal catalyzes luciferin to emit visible light, which can be visualized from outside the body. Transplanted islets from transgenic mice expressing luciferase under the control of the mouse insulin promoter could be visualized by bioluminescence, and the survival rate of islet grafts could be evaluated (45, 46). Although this method is a sensitive and quantitative measure of the beta-cell mass, it is not suitable for the clinical application.

Fluorescence imaging is also a useful modality for imaging pancreatic islets. Islets from transgenic mice expressing green fluorescent protein (47) or red fluorescent protein (48) under the control of the mouse insulin promoter could be imaged by a light signal generated from the light excitation to visualize islet engraftment. In addition, *ex vivo* fluorescence imaging of beta-cell apoptosis could be visualized after systemic administration of a fluorescent marker of apoptosis (CY5.5-labeled annexin V) (49). Furthermore, intravenous injection of a fluorescence-labeled exendin-4 analog with specificity for the GLP-1 receptor in beta-cells could distinguish beta-cells from exocrine pancreatic cells (50). However, these optical imaging modalities cannot visualize deep tissues and are restricted to preclinical studies.

## Conclusion and Future Expectations

**Figure 1:**
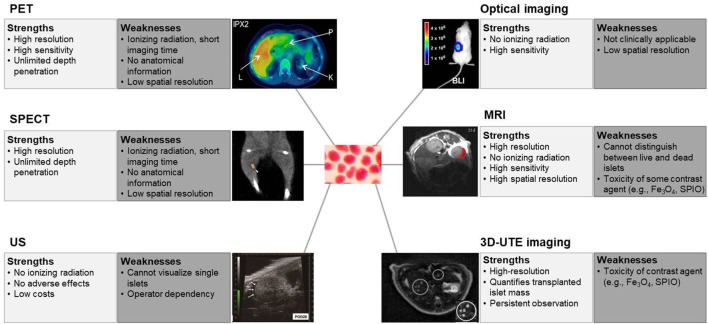
Strengths and weaknesses of non-invasive imaging of islets. The term “resolution” is used for a pixel count in digital imaging, and “spatial resolution” is used for the measure of how close lines can be resolved in an image, not just the pixel resolution in pixels per inch. PET, positron emission tomography. Reprinted with permission from Ref. (20). SPECT, single-photon emission computed tomography. Reprinted with permission from Ref. (25). US, ultrasonography. Reprinted with permission from Ref. (43). Optical imaging, especially bioluminescence imaging. Reprinted with permission from Ref. (46). MRI, magnetic resonance imaging. Reprinted with permission from Ref. (30). 3D-UTE, three-dimensional ultrashort echo time, imaging. Reprinted with permission from Ref. (42).

Pancreatic islet transplantation has a great potential for the treatment of type 1 diabetes. However, a significant number of transplanted islets are lost after transplantation, and therefore, the transplantation efficacy is insufficient to be a mainstream therapeutic method. It is difficult to investigate the cause of islet loss or intervention strategies to prevent islet loss without methods to assess islet survival or function. Current progress in non-invasive imaging techniques for transplanted islets, including PET, MRI, US, and optical imaging, allows quantification and functional evaluation of transplanted islets in experimental conditions. Techniques under development for imaging the pancreatic islets illustrate the capabilities and limitations of each imaging modality. The strengths and weaknesses of these imaging methods are summarized in Figure 1. Probes targeting biomarkers related to beta-cell function are another possibility for imaging, for example, Mn and Zn targeting (39, 40). MRI techniques might be possible for analysis of the functional beta-cell mass, because these images would reflect the insulin production of beta-cells. In addition, microencapsulated islets, rather than naked islets, would ameliorate the potential toxicity of labeling agents (51).

## Author Contributions

DK collected information; H-SJ collected information and wrote the manuscript.

## Conflict of Interest Statement

The authors declare that the research was conducted in the absence of any commercial or financial relationships that could be construed as a potential conflict of interest.
